# Enhancing Sustainability of Corroded RC Structures: Estimating Steel-to-Concrete Bond Strength with ANN and SVM Algorithms

**DOI:** 10.3390/ma15238295

**Published:** 2022-11-22

**Authors:** Rohan Singh, Harish Chandra Arora, Alireza Bahrami, Aman Kumar, Nishant Raj Kapoor, Krishna Kumar, Hardeep Singh Rai

**Affiliations:** 1 Department of Civil Engineering, Guru Nanak Dev Engineering College (GNDEC), Ludhiana 141006, India; 2 Department of Structural Engineering, CSIR-Central Building Research Institute, Roorkee 247667, India; 3 Academy of Scientific and Innovative Research (AcSIR), Ghaziabad 201002, India; 4 Department of Building Engineering, Energy Systems, and Sustainability Science, Faculty of Engineering and Sustainable Development, University of Gävle, 801 76 Gävle, Sweden; 5 Architecture and Planning Division, CSIR-Central Building Research Institute, Roorkee 247667, India; 6 Department of Hydro and Renewable Energy, Indian Institute of Technology, Roorkee 247667, India

**Keywords:** corrosion, bond strength, reinforced concrete, corroded steel reinforcement, machine learning, artificial neural network, support vector machine, sustainability

## Abstract

The bond strength between concrete and corroded steel reinforcement bar is one of the main responsible factors that affect the ultimate load-carrying capacity of reinforced concrete (RC) structures. Therefore, the prediction of accurate bond strength has become an important parameter for the safety measurements of RC structures. However, the analytical models are not enough to estimate the bond strength, as they are built using various assumptions and limited datasets. The machine learning (ML) techniques named artificial neural network (ANN) and support vector machine (SVM) have been used to estimate the bond strength between concrete and corroded steel reinforcement bar. The considered input parameters in this research are the surface area of the specimen, concrete cover, type of reinforcement bars, yield strength of reinforcement bars, concrete compressive strength, diameter of reinforcement bars, bond length, water/cement ratio, and corrosion level of reinforcement bars. These parameters were used to build the ANN and SVM models. The reliability of the developed ANN and SVM models have been compared with twenty analytical models. Moreover, the analyzed results revealed that the precision and efficiency of the ANN and SVM models are higher compared with the analytical models. The radar plot and Taylor diagrams have also been utilized to show the graphical representation of the best-fitted model. The proposed ANN model has the best precision and reliability compared with the SVM model, with a correlation coefficient of 0.99, mean absolute error of 1.091 MPa, and root mean square error of 1.495 MPa. Researchers and designers can apply the developed ANN model to precisely estimate the steel-to-concrete bond strength.

## 1. Introduction

The maintenance and retrofitting of reinforced concrete (RC) structures have become critical issues in the civil engineering sector for having great serviceability and large ultimate load-carrying capacity. Deterioration of concrete structures mainly happens due to the lack of maintenance, environmental decay, reinforcement corrosion, and aging of structures through time [1]. The gradual decomposition of objects, usually made of metal, brought on by the environment and chemical reactions is referred to as corrosion. One of the best examples of this phenomenon is iron oxide formation or rusting. Alkaline solution is found in the pores of the hydrated cement paste, which supports passivation and defends steel in concrete. Further, there is a formation of a thin layer of oxide film on the steel surface that protects it from corrosion. However, when this thin layer of an oxide film is locally damaged or removed, corrosion may occur [2]. Chloride penetration or carbonation of concrete may be responsible for the removal of an oxide film. The pH of fresh concrete is in the range of around 12 to 13, but decreases significantly to 8.5 when it reacts with carbon dioxide in the air. The process of carbonation results in the deterioration of RC. In the presence of moisture, carbon dioxide transforms into diluted carbonic acid, which destroys concrete and lowers its alkalinity. The types of corrosion include sulfidic corrosion (sulfidation), uniform corrosion, and localized corrosion. Pitting corrosion, galvanic corrosion, crevice corrosion, and selective weld attack all fall into the category of localized corrosion.

Corrosion of the reinforcement bars embedded in concrete is regarded as the primary cause of deterioration of RC structures and may result in serious damage to RC structures [3]. Particularly when the structural service life increases, corrosion will cause the link between concrete and reinforcement bar to erode, which lowers the structural performance and dependability [4]. The corrosion of reinforcement bar affects reinforcing by reducing the reinforcement bar diameter, and influences concrete by cracking due to the volumetric expansion of the corrosion products. Hence, it affects the interaction between the reinforcement bar and concrete due to the loss of bond between them.

For all RC structures, the bond between steel-to-concrete is an important parameter. Transferring loads between concrete and reinforcement bar is necessary to maintain the composite action. This load transfer is referred to as a bond. More bond strength (BS) between concrete and steel leads to safer and easier transfer of the load [5]. BS is directly proportional to the load-carrying capacity of RC structures. The main factors that affect BS between concrete and reinforcement bar are concrete compressive strength (CS), concrete cover, the diameter of reinforcement bars, type of reinforcement bars, and spacing between reinforcement bars. It is crucial to evaluate the loss of steel-to-concrete BS for the calculation of the remaining residual capacity of RC elements impacted by corrosion of reinforcement bar. Once the structure’s remaining bond capacity is determined, the residual service life might be found in the corrosion-affected RC structures [6,7]. The general formula to find BS is given below:

(1) $$\tau_{u}=\frac{P_{F}}{\pi \times d\times L_{b}}$$

where *P_F_* is the pullout force (kN), *d* is the diameter of the reinforcement bar (mm), and *L_b_* is the bond length (mm).

Most of the studies available in the literature have shown that BS between reinforcement bar and concrete is slightly increased to some extent of the corrosion level. In most of the studies, an accelerated corrosion process was applied for the prediction of BS between corroded reinforcement bar and concrete. In the experimental study carried out by Coccia et al. [8] to determine the influence of corrosion on BS of steel-to-concrete, the results demonstrated that low corrosion percent (lower than 0.6) led to an increase of BS (50–60%) and higher corrosion entities caused a sharp bond reduction. Another research was conducted by Yalciner et al. [9] to evaluate BS of steel-to-concrete as a function of concrete cover, CS of concrete, and level of corrosion. The experimental results illustrated that the degradation of BS is more in high-strength concrete compared with low-strength concrete. Chung et al. [10] assessed the bond behavior of highly corroded reinforcement bars and analyzed results to reveal that BS increases up to maximum value but eventually decreases for greater corrosion values. Ma et al. [11] determined the effect of corrosion on the steel-to-concrete BS. The results depicted that the bond behavior between the deformed reinforcement bar and concrete was less sensitive to corrosion than smooth reinforcement bars. Choi et al. [12] examined the effect of corrosion on the bond characteristics in RC specimens. The experimental results indicated that the slip at failure was increased when the corrosion level was higher than 5%, and there was an increase in BS when the corrosion level was lower than 1%; also, a brittle failure pattern was observed when the area of corrosion exceeded 50%.

For the estimation of the bond behavior of corroded RC, many mechanically driven empirical models are available in the literature. However, these models have only a limited degree of accuracy since the hidden mechanism is so complicated. Additionally, although the majority of models are deterministic, the problem itself is probabilistic since there are a variety of uncertainties, including uncertainty related to material qualities and geometric dimensions [4].

To create a complete and comprehensive dataset, the current research work has collected the experimental BS data of corroded reinforcement bar (without any type of stirrups) from previously published studies. Twenty analytical models have also been gathered to check the precision of the machine learning (ML) model. The ML-based artificial neural network (ANN) and support vector machine (SVM) models have been developed to predict the steel-to-concrete BS. The ANN algorithm is used as an effective ML technique and precise estimation approach of BS between concrete and corroded reinforcement bar. This study demonstrates that the developed ANN has enough capabilities to predict steel-to-concrete BS.

### 1.1. Determination of BS

**Figure 1:**
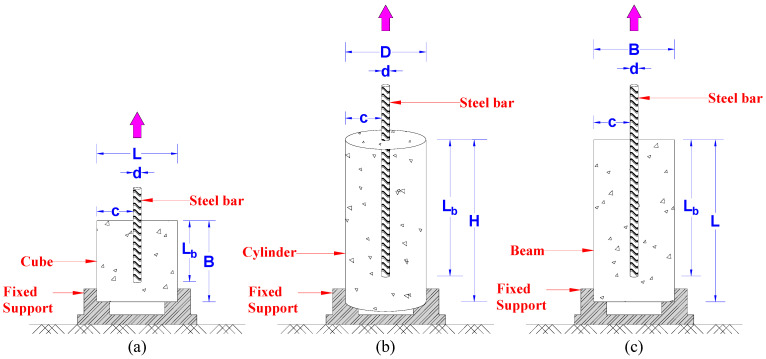
Pullout test setup; (**a**) cube, (**b**) cylinder, (**c**) beam specimens.

The pullout test is mainly used to determine the steel-to-concrete BS (cubes, cylinders, and beams). As indicated in Figure 1, the test specimen must be installed in a suitable testing apparatus so that the reinforcement bar is pulled axially from the specimen. Figure 1a–c shows the setup for the cube, cylinder, and beam specimens, sequentially. The pullout testing procedure is detailed in the published literature [8,9,10,12].

The maintenance of RC structures is one of the major activities of the civil engineering industry. Corrosion has a large influence on the bond, shear, and flexural strength; hence, it is crucial to estimate how much bond capacity remains after the structure has been affected by corrosion. Accurate prediction of BS helps assess the residual life, structural performance, and reliability of RC structures. It is essential to utilize a precise and effective model for predicting BS of RC in order to increase the residual life of structures. The computational-based models can achieve higher accuracy with minimal errors. The developed ANN model in this study is the most suitable and practically applicable model with high precision. The proposed ANN model also outperforms the existing ML as well as analytical models. The results of this study provide a practically applicable model to estimate the steel-to-concrete BS. The proposed ML model is more accurate and can help researchers and designers precisely predict BS with less experimental effort.

## 2. Application of ML in Concrete Technology

The development of data mining and ML techniques over the past few decades has made it possible to create prediction models from empirical data without having a thorough understanding of the underlying physical principles. As a result, these models may be useful for predicting various properties of concrete as well as numerous forces acting on structural members that can be impacted by compositional, processing, and testing factors.

Soft computing approaches have effectively been used in recent years to predict several important properties of RC structures and other different engineering applications [13,14,15,16]. ML algorithms are suitable for solving concrete-like complex problems and developing good relationships between input and output parameters. Nowadays, ANN is employed for estimating universal concerns in numerical models because of their eminent properties to self-learn, adapt, and tolerate faults.

The previous studies that applied the ML algorithms to find BS are summarized below and mentioned in Table 1. Concha and Oreta [17] did the estimation of BS with the ANN model. The collected dataset contains 108 concrete cube samples (BS in the range of 6.029 to 30.922 MPa), and only four input parameters were considered to estimate BS. The results illustrated that the correlation coefficient (R) value of the ANN model was 0.927. Mousavi et al. [7] estimated BS with three ML models, namely MLP, RBFNN, and SVR, with 482 experimental datasets, while the BS range was 0.19 to 91.42 MPa. The considered input parameters were eight, and the analyzed results displayed that the precision of the SVR (R = 0.977) model was higher compared with other ML models. Rahman and Al-Ameri [18] used the ANN algorithm to estimate the steel-to-concrete BS. Only twenty-one experimental datasets were employed to develop the ML model, and the BS range was between 2.32 to 14.15 MPa. It was concluded that the accuracy of the ANN model was good, with an R-value of 0.963.

Farouk et al. [19] estimated BS with multiple linear regression (MLR), SVR, ANN, PSO, IEPSO, PANN, and IEPANN algorithms. Among all the ML models, the precision of the IEPANN model was good, with an R-value of 0.973. ANFIS, ANN, and GMDH were utilized by Alizadeh et al. [20] to estimate (1.64 to 22.34 MPa) with 159 experimental datasets. The results depicted that the reliability of the ANFIS model was higher, with an R-value of 0.988 compared with other ML models. Amin et al. [21] applied gene expression programming (GEP) to estimate BS in the range of 0.76 to 21 MPa. The R-value of the GEP model was 0.963 and showed sufficient accuracy of the developed model. The R-value of the developed model was 0.945. MLP, RBFNN, and GEP models were applied by Ben Seghier et al. [22] to estimate BS. The collected dataset contained 218 experimental values that had BS in the range of 1.3 to 31.7 MPa. The precision of the MLP-LMA (R = 0.97) model was higher than the rest of the ML models. Yartsev et al. [23] utilized only the ANN model with 250 experimental datasets, with BS in the range of 1.3 to 31.7 MPa; the R-value of the developed ANN model was 0.947. Bseiso [24] used the ANN algorithm with two different activation functions (AF), namely sigmoid and rectified linear unit (ReLU), on the collected database of 90 experimental values with a BS range of 1.3 to 31.7 MPa. ANN with ReLU activation revealed a higher R-value of 0.983 with higher accuracy. Kumar et al. [25] studied BS between fiber-reinforced cementitious mortar and concrete surfaces with 10 different machine-learning models. Among all the models, the precision of the optimized GPR model was good, with an R-value of 0.9336.

## 3. Experimental Data and Methods

### 3.1. Data Bank

In this research, 476 experimental datasets of corroded steel-to-concrete BS were collected from previous studies. All the experimental data were of the pullout test without any stirrups or transverse reinforcement bars provided [8,9,10,11,12,35,36,37,38,39,40,41,42,43]. This study took nine input parameters into account; surface area of the specimen (S_AS_), water/cement ratio (*w/c*), CS of concrete (*f ′_c_*), concrete cover (*c*), bond length (*L_b_*), diameter of reinforcement bars (*d*), type of reinforcement bars (*T_b_*), yield strength of reinforcement bars (*f_y_*), and corrosion level of reinforcement bars (*η*) to predict BS (*τ_u_*), which represents the output parameter. Table 2 lists the details of the collected database that includes the series of input and output parameters. The range of BS in the database was from 0.19 to 91.42 MPa and the corrosion level varied from 0 to 22.90%. Table 3 presents the statistical parameters of the collected database, such as mean, minimum, maximum, and standard deviation (Std.) values.

**Figure 2:**
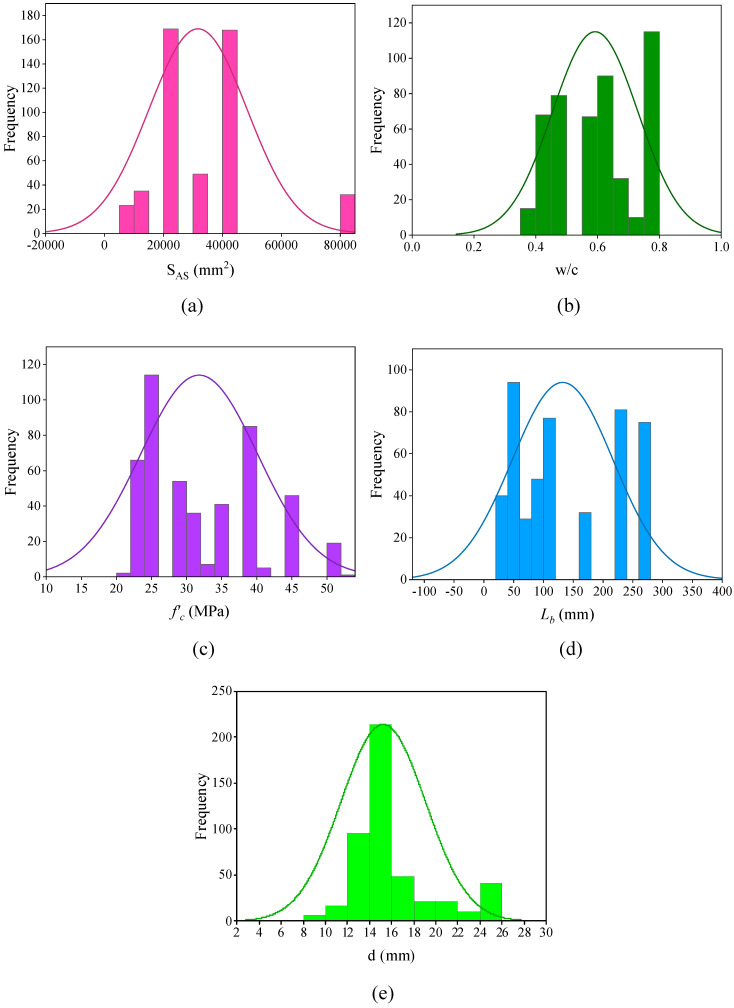

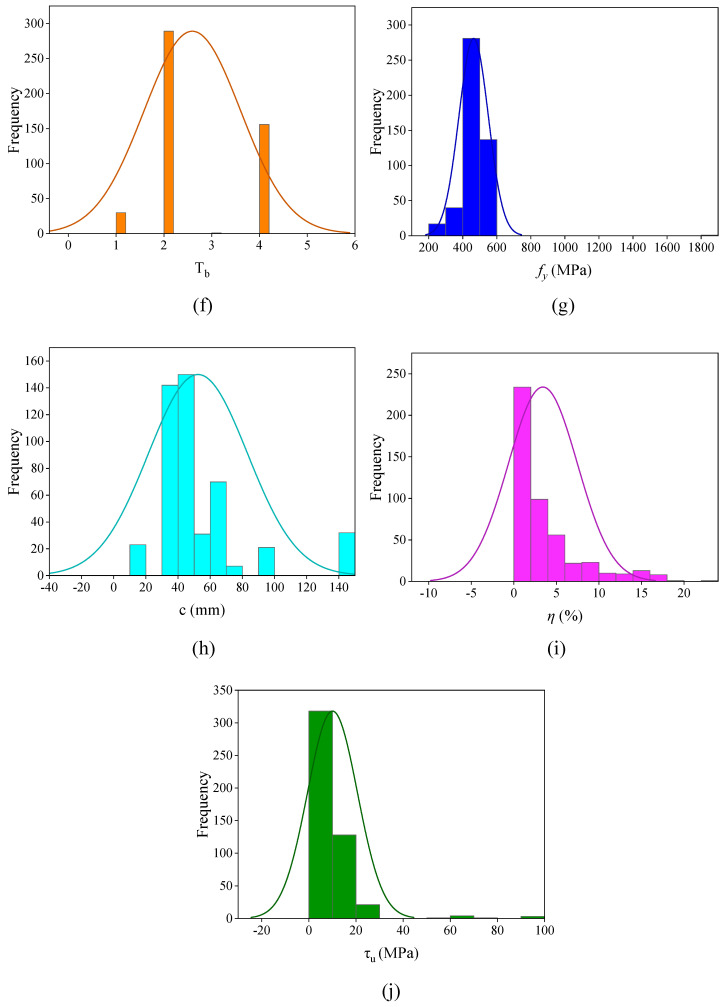
Relative frequency distribution of input and output parameters; (**a**) *S_AS_*, (**b**) *w/c*, (**c**) *f ^’^_c_*, (**d**) *L_b_*, (**e**) *d*, (**f**) *T_b_*, (**g**) *f_y_*, (**h**) *c*, (**i**) *η*, (**j**) *τ_u_*.

**Figure 3:**
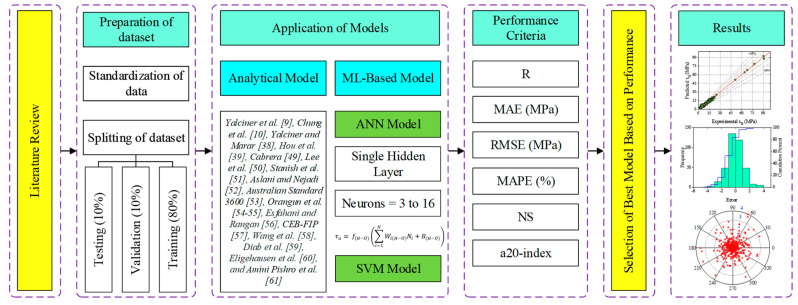
Methodology diagram for proposed work.

The relative frequency distribution of input and output parameters is illustrated in Figure 2. The maximum used values of *S_AS_* for most of the dataset were between 20,000 and 40,000 mm^2^. The majority of data have used a w/c range of 0.75 to 0.8. The most frequently used value for CS of concrete was 25 MPa. Many of the collected specimens had an embedded length of 50 mm with a 14–16 mm reinforcement bar diameter. In most of the cases, a deformed reinforcement bar was used in the specimens, as shown in the graph. Most frequently, steel reinforcement bars with *f_y_* between 400 and 500 MPa were employed. The range of *c* was 30 to 50 mm. The majority of the specimens had a corrosion level between 0 and 2%. BS values of many of the specimens were in the range of 1 to 10 MPa. The methodology adopted to achieve the objective of the current study is reported in Figure 3.

### 3.2. Preparation of Data

Preparation of data is a process of formatting, transforming, or checking the accuracy of data. After collecting the experimental data from the previous studies, the process of data normalization must be done before operating the ML algorithms. Data normalization helps scale the data so that all the numeric values come under the same range. This issue increases the readability of the data by the ML algorithms and also helps improve the model accuracy, enhance the generalization ability, and learning ability of the algorithms. As the parameters of the data are in different units, therefore, it is essential to make the data unit less. The data have been standardized in the range of −1 to +1 using Equation (2) [44].

(2) $$z_{n\;}=\left[2\times \frac{\left(z-z_{min}\right)\;}{\left(z_{max}-z_{min}\right)}\right]-1$$

where *z_n_* is the normalized output of variable *z*, *z* is the variable of input to be normalized, and *z_max_* and *z_min_* are the maximum and minimum values of the input variable *z*, respectively.

After the process of normalization, the dataset was divided into three categories, in which 80% of the datasets were randomly selected for training, 10% were selected for testing, and 10% were selected for validation (as shown in Figure 3). Hence, 476 specimens were distributed as 380 (for training), 48 (for testing), and 48 datasets (for validation).

### 3.3. Performance Criterion

Performance indices were used to assess the efficacy and performance of the analytical as well as the ML models. R, a20-index, mean absolute error (MAE), root mean square error (RMSE), mean absolute percentage error (MAPE), and Nash–Sutcliffe efficiency index (NS), are the most used performance metrics. The MAPE, MAE, and RMSE represent the errors, and lower the values of these errors resulting in a higher R, and thus, the accuracy and performance of the ML models will be greater [45,46]. If the values of R, NS, and a20-index are near 1, then it reveals a strong positive relationship between the models. The mathematical expression of these indices is given below [47,48]:

(3) $$R=\frac{\sum_{i=1}^{N}\left(r_{i}-\bar{r}\right)\left(s_{i}-\bar{s}\right)}{\sqrt{\sum_{i=1}^{N}{\left(r_{i}-\bar{r}\right)}^{2}\sum_{i=1}^{N}{\left(s_{i}-\bar{s}\right)}^{2}}}$$

(4) $$MAE=\frac{1}{N}\overset{N}{\underset{i=1}{\sum }}\left|r_{i}-s_{i}\right|$$

(5) $$RMSE=\sqrt{\frac{1}{N}\overset{N}{\underset{i=1}{\sum }}{\left(r_{i}-s_{i}\right)}^{2}}$$

(6) $$MAPE=\frac{1}{N}\overset{N}{\underset{i=1}{\sum }}\left|\frac{r_{i}-s_{i}}{r_{i}}\right|\times 100$$

(7) $$NS=1-\frac{\sum_{i=1}^{N}{\left(r_{i}-s_{i}\right)}^{2}}{\sum_{i=1}^{N}{\left(r_{i}-\bar{s}\right)}^{2}}$$

(8) $$a20-index=\frac{m20}{N}$$

where *r* and *s* are the experimental and predicted output sets, respectively, while $\bar{r}$ and $\bar{s}$ are the means of the experimental and predicted output sets, respectively. *N* is the number of points in the dataset, and *m*20 is the number of values obtained from measured/predicted values and falls into the range of 0.8 to 1.2 [1].

### 3.4. Analytical Models

Twenty analytical models were collected from the literature and used to assess the accuracy of the developed ANN model. Most of the commonly used available analytical models that were utilized to estimate the steel-to-concrete BS are summarized in Table 4. For easy understanding and further analysis, the collected analytical models are assigned different model identities. Cabrera [49], Lee et al. [50], Stanish et al. [51], Chung et al. [10], Aslani and Nejadi [52], Yalciner et al. [9], Yalciner and Marar [38], Australian Standard 3600 [53], Orangun et al. [54,55], Esfahani and Rangan [56], CEB-FIP [57], Hou et al. [39], Wang et al. [58], Diab et al. [59], Eligehausen et al. [60], and Amini Pishro et al. [61] are assigned to Models 1–20.

## 4. ML

### 4.1. SVM

Vapnik and Chervonenkis created the SVM algorithm in 1963, and it was originally presented by Boser, Guyon, and Vapnik at the Computational Learning Theory conference in 1992 [62]. SVM is a type of ML algorithm that comes under the category of supervised learning and is used to analyze data for classification, prediction, and regression analysis [7]. However, mostly, it is used to analyze data for classification problems in ML. The working of SVM is based on the principle of statistical learning theory and structural risk minimization (SRM) to attain good generalization performance. SVM is a data-driven algorithm that forms a relationship between the input data and the target-dependent variable in accordance with the principle of SRM [34]. For easy placing of new data points in the correct category, the SVM algorithm’s objective is to establish a boundary that can divide N-dimensional space into groups or classes, and this best line boundary is known as the hyper plane. There will be many decision boundaries, but for best results, the best decision boundary should be selected (the boundary with the maximum distance between data points), and the main goal of the SVM algorithm is to maximize the margin between the data points. Support vectors are the extreme points or vectors that contribute to the creation of the hyper plane, which are selected by SVM.

Some of the applications of SVM include the classification of images, face detection, text and hypertext categorization, and bioinformatics. The advantages of SVM are high dimensional input space, regularization parameters, and sparse document vectors. Numerous scholars have used the SVM approach to great success in the field of structural engineering.

Considering a set of training points {(*x*_1_, *y*_1_) …… (*x_i_*, *y_i_*)}, where the following function helps define the nonlinear mapping between input and output vectors, *xi* ∈ RN is an N-dimensional input vector and *y_i_* ∈ *R* is the target output. The predicted value, *f*(*x_i_*)**,** can be calculated from Equation (9):

(9) $$f\left(x_{i}\right)=\omega^{T}\phi \left(x_{i}\right)+b$$

where *ω* and *b* are the support vector variable and bias, respectively, and *ϕ*(*x_i_*) is the transfer function (nonlinear mapping function).

For the training dataset with l sample, the standard form of the ϑ-SVM optimization model can be expressed as follows [44]:

(10) $$\{\begin{array}{c} \min R\left(\omega ,\xi ,\xi^{*},\;\varepsilon \right)=\frac{1}{2}{∥\omega ∥}^{2}+C\left[\vartheta \varepsilon +\frac{1}{l}\overset{l}{\underset{i=1}{\sum }}\left(\xi_{i}+\xi_{i}^{*}\right)\right]\\ subject\;to:\;y_{i}-\omega^{T}\phi \left(x_{i}\right)-b\leq \varepsilon +\xi_{i}\\ \frac{\omega^{T}\phi \left(x_{i}\right)+b-y_{i}\leq \varepsilon +\xi_{i}}{\xi^{*},\varepsilon \geq 0} \end{array}$$

where *C* is the variable used to stabilize the model difficulty and experiential risk term ${∥\omega ∥}^{2}$, and $\xi_{i}^{*}$ denotes the distance and is called a slack variable. To solve dual optimization problems, the Lagrange technique of multipliers is used in the following equation [44].

(11) $$\{\begin{array}{c} \max R\;\left(a_{i},\;a_{i}^{*}\right)=\overset{l}{\underset{i=1}{\sum }}y_{i}\left(a_{i},a_{i}^{*}\right)-\frac{1}{2}\overset{l}{\underset{i=1}{\sum }}\overset{l}{\underset{j=1}{\sum }}\left(a_{i}-a_{i}^{*}\right)\left(a_{j}-a_{j}^{*}\right)K\left(x_{i},x_{i}^{*}\right)\\ subject\;to:\;\overset{l}{\underset{i=1}{\sum }}\left(a_{i}-a_{i}^{*}\right)=0,\;\;0\leq a_{i},a_{i}^{*}\leq C/l\\ \overset{l}{\underset{i=1}{\sum }}\left(a_{i}+a_{i}^{*}\right)\leq C.v \end{array}$$

where kernel functions are denoted by *K* (*x_i_*, *x_j_*), and the non-negative Lagrange multipliers are represented by a_i_ and a_i_^*^.

Equation (12) states the regression for an unknown input vector *x*,

(12) $$f\left(x\right)=\overset{l}{\underset{i=1}{\sum }}\left(a_{i}-a_{i}^{*}\right)\times K\left(x,x_{i}\right)+b$$

where $K\left(x,x_{i}\right)$ is a kernel function, and $\left(a_{i}-a_{i}^{*}\right)$ and *b* are the results to the problem.

### 4.2. ANN

In 1956, John McCarthy first coined the term “Artificial Intelligence” at the Dartmouth conference. Artificial intelligence is like mimicking human behavior. A computer system that can simulate human intellect is created via the science of artificial intelligence. The history of ANN began with Warren McCulloch and Walter Pitts in 1943 when they proposed the first mathematical model inspired by biological neurons, resulting in the first conception of the artificial neuron. A technology known as ANN was developed using research on the nervous system and brain [63]. ANN is a special kind of ML algorithm that is similar to the neurons in the human brain. In general, multiple neurons come together to form an ANN, which works as the foundation for the operation of a function in accordance with its task [64]. ANN has been used for nonlinear modeling in a variety of engineering fields, including ocean engineering, hydraulics, and geotechnical engineering.

The signals or samples that reflect the values supposed by the variables of a certain application are known as “input signals”. To improve the computing efficiency of learning algorithms, the input signals are generally normalized. Each input variable is given a weight, called a synaptic weight, which allows the relevance of each one to the neuron’s functionality to be quantified. The variable known as the bias (activation threshold) is used to define the appropriate threshold that the output of the linear aggregator should have to provide a trigger value for the neuron output. The purpose of AF, which is predicated by its functional picture, is to limit the neuron output within a suitable range of values. The output signal is the final value that a neuron produces in response to a specific set of input signals. It can also serve as an input for further neurons that are sequentially connected [63].

**Figure 4:**
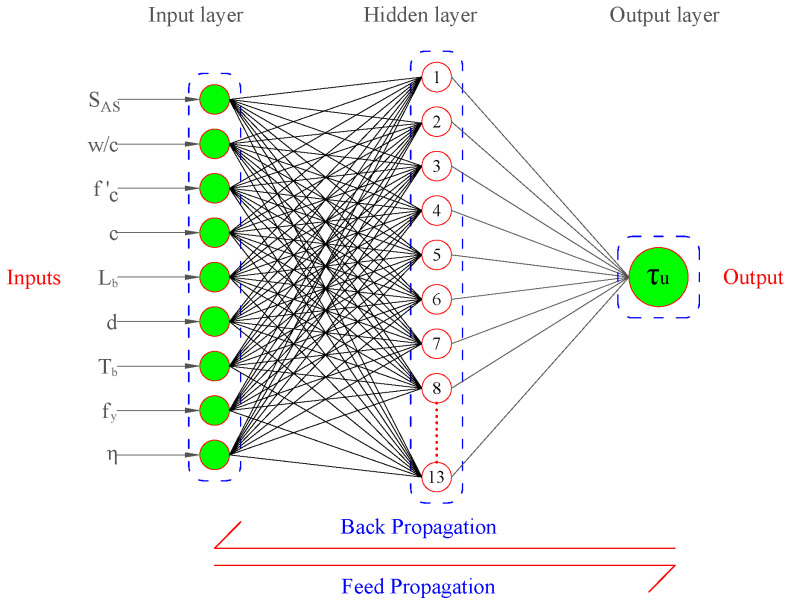
Architectural diagram of ANN.

An ANN can generally be divided into three groups called input, hidden (intermediate), and output layers, as displayed in Figure 4. The input layer has the responsibility of collecting the data (information), signals, and characteristics from the external domain. The hidden layer is made up of neurons that are in charge of identifying patterns connected to the system or process under study. Most of the network’s internal processing are carried out by these layers.

The following categories can be used to group the basic architectures of ANN: (i) single-layer feed-forward networks, (ii) multilayer feed-forward networks, (iii) recurrent networks (feedback architecture), and (iv) mesh networks. In the ML field, the word “epoch” refers to the total number of runs the algorithm has made across the whole training dataset [63].

#### Development of ANN Model

An ANN network with three layers as input, hidden, and output was developed in order to produce an ML model that could predict the corroded steel-to-concrete BS. Further, the input layer was composed of input nodes known as the input parameters, the hidden layer was composed of neurons that help receive the data from the input layer, process the data, and produce the desired output. However, the output layer was composed of the target parameter. The complexity of the network increases with an increase in the number of hidden layers. The development of an ANN model with the minimum number of layers is always a better approach. In this study, single hidden layer was used to develop the reliable and easy-to-use ANN model.

In order to increase the effectiveness of the training procedure, the Levenberg-Marquardt (LM) algorithm, a second-order gradient algorithm based on the least-squares method for nonlinear models, was applied to the backpropagation algorithm [63].

This work also makes use of the LM algorithm, one of the most effective training algorithms. The LM procedure randomly divides input and output vectors of data into three categories: training, testing, and validation. In nonlinear least squares problems, the LM method is an iterative process that is commonly used as a learning technique [65].

AF is the function that is used to obtain the output of the node; it is also known as the transfer function. It maps the resulting values between 0 to 1 or −1 to 1, etc., depending upon the function. The most common AFs are “TanSig” and “LogSig”. In this research work, the “TanSig” function was applied between the input to the hidden layer. The mathematical equation for the “TanSig” function is expressed as:

(13) $$TanSig=\frac{2}{1-e^{-2z}}-1$$

where *z* represents the value of the input. Other various AF are used in the ANN, such as linear function, nonlinear function, Gaussian function, hyperbolic tangent function, Sigmoid or ReLU, logistic function, and leaky ReLU.

**Figure 5:**
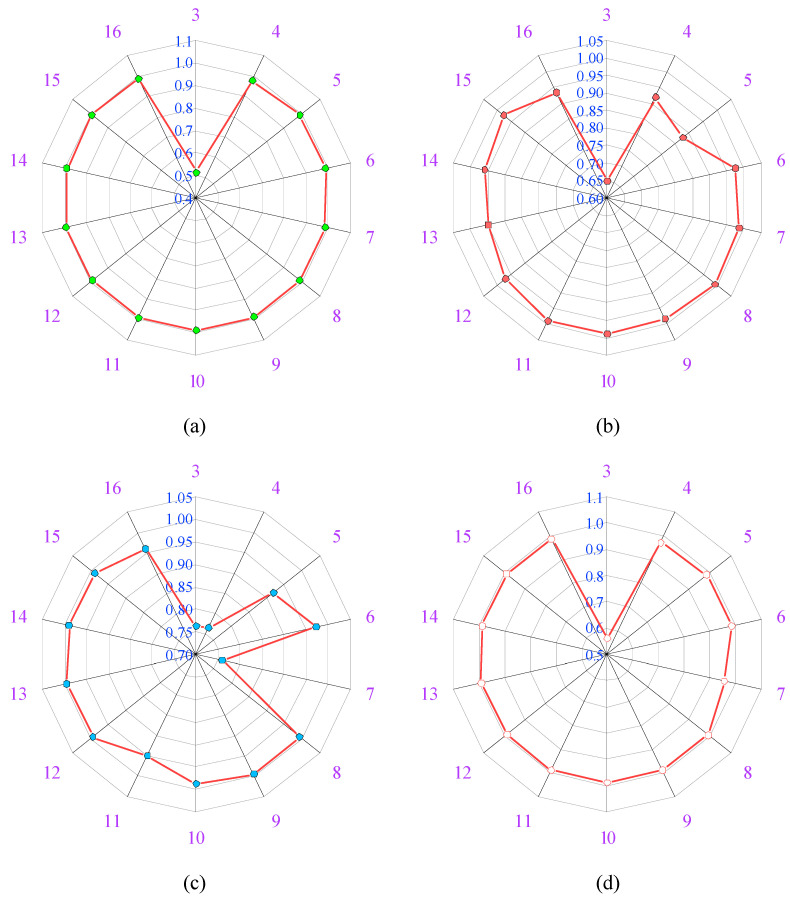
Values of R for each neuron; (**a**) training dataset, (**b**) validation dataset, (**c**) testing dataset, (**d**) overall dataset.

**Figure 6:**
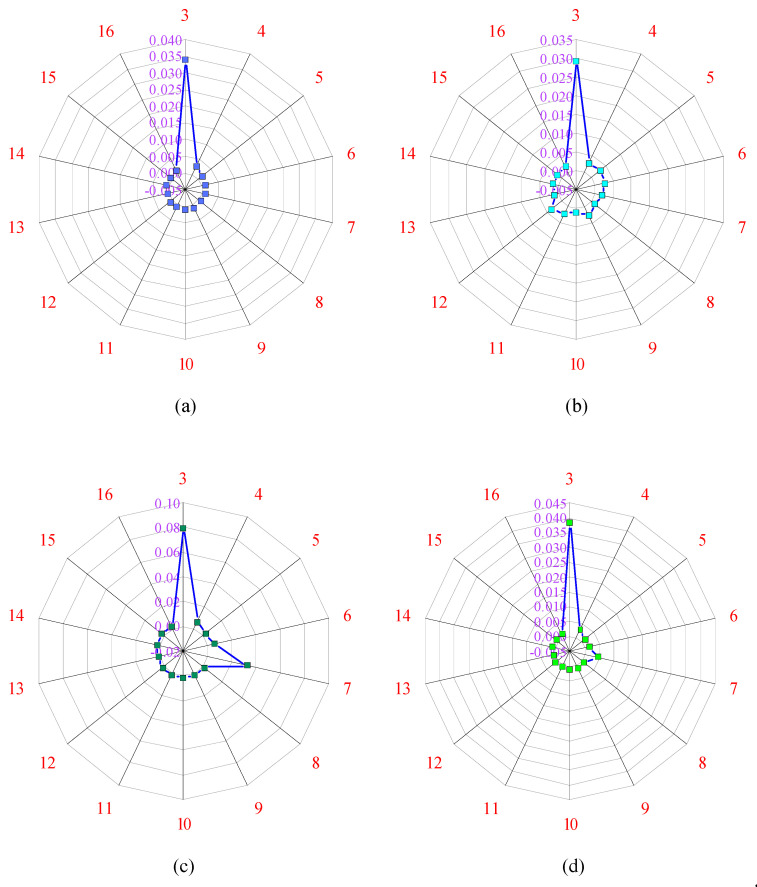
Values of MSE for each neuron; (**a**) training dataset, (**b**) validation dataset, (**c**) testing dataset, (**d**) overall dataset.

By applying a trial-and-error approach to obtain optimum performance, the percentages of training, testing, and validation set are considered as 80%, 10%, and 10%, respectively. Afterward, the hit and trial method was applied, in which hidden layer neurons were trained by changing the number of neurons from 3 to 16. The performance of each neuron was calculated by the ranking of neurons based on R and MSE values, as depicted in Figure 5 and Figure 6, respectively. The process of training the data was carried out by changing the number of neurons, the neurons which indicated the best performance was taken as the final result. All the performance indices were calculated after selecting the best neuron. This work makes use of a linear transfer function named as “purelin” function from the hidden to the output layer. After that, all the values of biases and weights between layers are determined, and then AF is applied to get the output [6].

The R-value of each neuron is provided in Figure 5. As illustrated in Figure 5, neuron 13 has the maximum R-value of the overall dataset close to one. Similarly, as shown in Figure 6, the MSE value at neuron 13 is minimum and approaches zero. As a result, the network performed at its optimum level when the hidden layer contained 13 neurons. The steel-to-concrete BS can be calculated using the equations below:

(14) $$N_{i}=f_{\left(I-H\right)}\left(\overset{N}{\underset{i=1}{\sum }}W_{i\left(I-H\right)}X_{i}+B_{\left(I-H\right)}\right)$$

where $W_{i\left(I-H\right)}$ is the value of weights from input to the hidden layer, $X_{i}$ is the normalized input value, $B_{\left(I-H\right)}$ is the value of bias from input to hidden layer, and $f_{\left(I-H\right)}$ is AF that is used from input to hidden layer (“TanSig” transfer function is used), and $N_{i}$ is the input parameter that is the sum of biases, weights, and normalized inputs. The final output of the ANN model (BS), $\tau_{u}$, can be achieved from Equation (15):

(15) $$\tau_{u}=f_{\left(H-O\right)}\left(\overset{N}{\underset{i=1}{\sum }}W_{i\left(H-O\right)}N_{i}+B_{\left(H-O\right)}\right)$$

where $W_{i\left(H-O\right)}$ is the value of weights from the hidden to the output layer, $B_{\left(H-O\right)}$ is the value of bias from the hidden layer to the output layer, and $\;f_{\left(H-O\right)}$ is AF that is used from the hidden to the output layer (“purelin” transfer function is used), and $N_{i}$ is the value that is obtained from Equation (10).

## 5. Results and Discussion

In this section, the results of the analytical models and ANN models are explained individually. The comparison of the analytical results with the ANN model is summarized in the discussion section. A Taylor plot is used to demonstrate the graphical fitting of the analytical and developed ANN models. The ANN mathematical formulation is also expressed in the current section.

### 5.1. Results of Analytical Models

**Figure 7:**
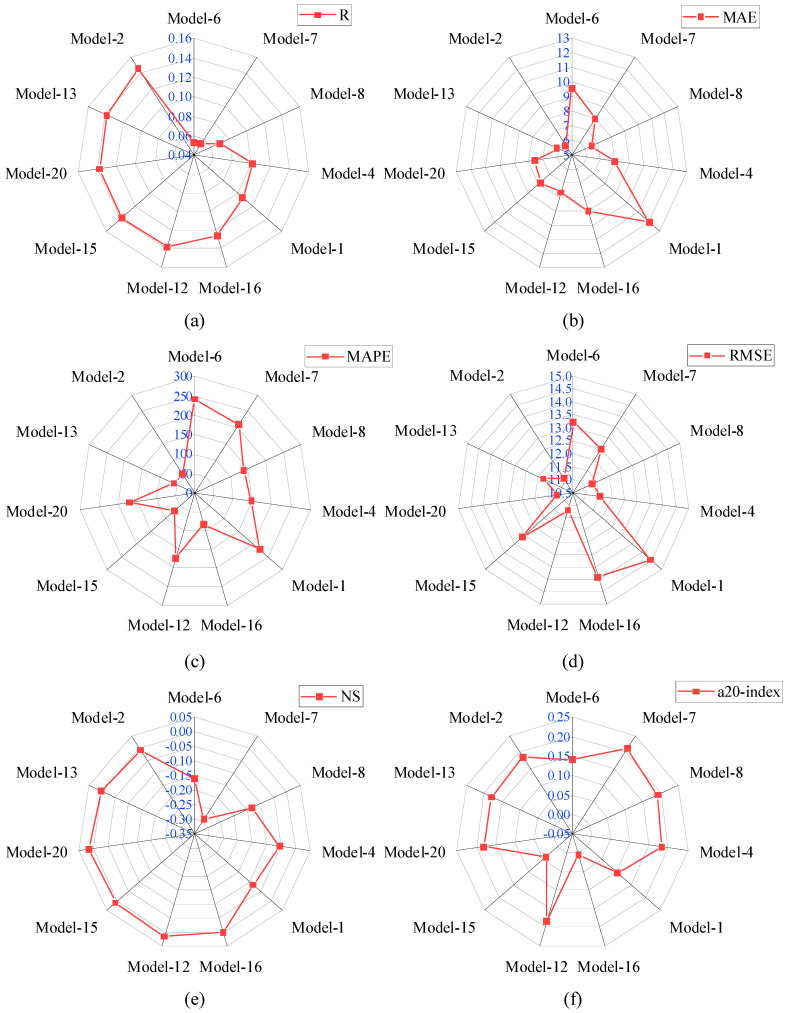
Comparison of performance indices of Group-I models; (**a**) R, (**b**) MAE, (**c**) MAPE, (**d**) RMSE, (**e**) NS, (**f**) a-20 index.

The selected input parameters have been processed through twenty analytical models. The details of these analytical models are available in Section 4. For better understanding, the analytical models have been divided into three groups based on the R-values. The range of the R-values for Group-I, II, and III were 0–0.15, 0.15–0.30, and 0.30–0.40, respectively. The other performance indices, such as MAE, RMSE, MAPE, NS, and a20-index have also been used to assess the performance of these analytical models. The R-values of Model-6, Model-7, Model-8, Model-4, Model-1, Model-16, Model-12, Model-15, Model-20, Model-13, and Model-2 were 0.053, 0.054, 0.069, 0.101, 0.107, 0.126, 0.138, 0.138, 0.138, 0.139, and 0.139, respectively. The MAE value of Model-1 was 12.036 MPa, which was the highest among Group-I models. Similarly, the RMSE value of Model-1 was the highest, as well. However, the MAPE value of Model-6 was the highest, which was 241.66%. The a20-index and NS values of Model-7 and Model-20 were highest in Group-I models. Based on all the performance indices, the reliability of Model-2 was good compared with other Group-I models. The R-value of Model-2 was 175.47%, 170.37%, 111.59%, 44.55%, 36.45%, 15.87%, and 5.04% higher than Model-6, Model-7, Model-8, Model-4, Model-1, Model-16, and Model-13, respectively. The comparison of the performance indices of Group-I models is presented in Figure 7.

**Figure 8:**
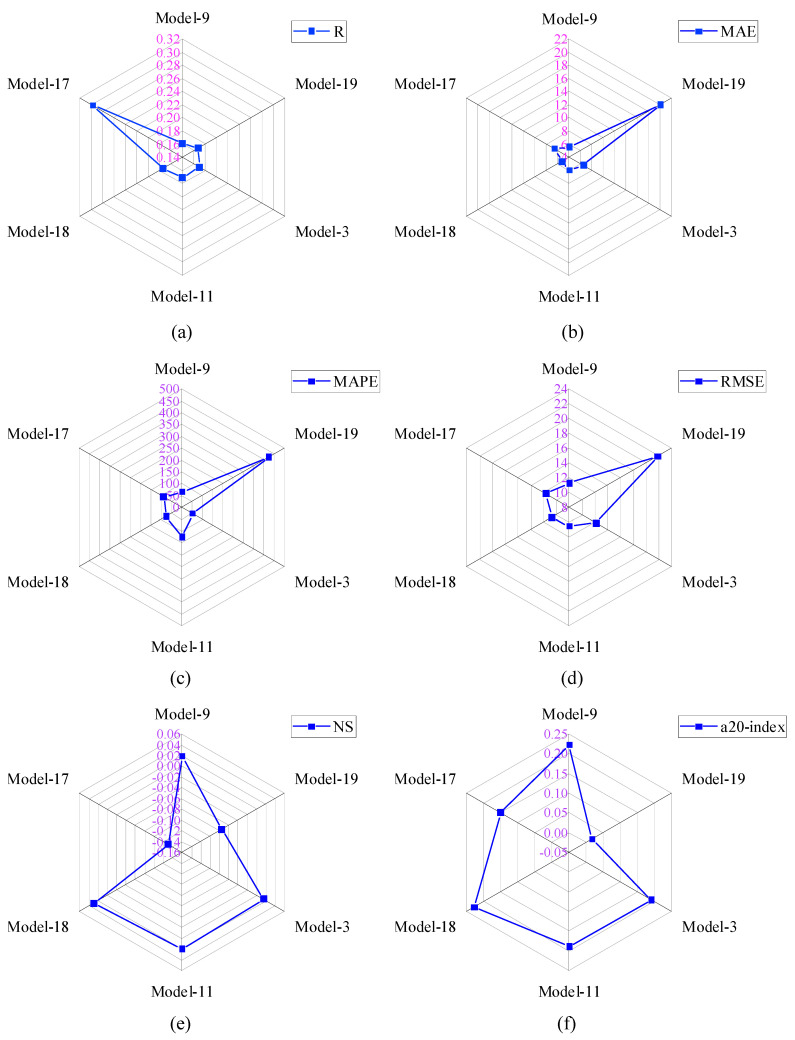
Comparison of performance indices of Group-II models; (**a**) R, (**b**) MAE, (**c**) MAPE, (**d**) RMSE, (**e**) NS, (**f**) a-20 index.

The R-value of Model-2 was 5.8% higher than Model-12, Model-15, and Model-20, sequentially. The MAE value of Model-2 was the lowest among Group-I models. The MAE value of Model-2 was 39.58%, 27.23%, 10.99%, 27.29%, 51.91%, 35.75%, 24.34%, 26.47%, 23.34%, 5.55% lower than Model-6, Model-7, Model-8, Model-4, Model-1, Model-16, Model-12, Model-15, Model-20, and Model-13, respectively. The comparison of the performance indices of Group-II models is demonstrated in Figure 8.

In Group-II models, the R-values were between 0.15 and 0.30. Only six analytical models fell into the group. The R-values of Model-9, Model-19, Model-3, Model-11, Model-18, and Model-17 were 0.160, 0.168, 0.171, 0.171, 0.174, and 0.298, sequentially. The R-value of Model-17 was the highest among Group-II models, which was 86.25%, 77.38%, and 71.26% higher than Model-9, Model-19, and Model-17, respectively. Similarly, the R-value of Model-17 was 74.27% higher than Model-3 and Model-11. The MAE value of Model-18 was the lowest one compared with other Group-II models. This MAE value was 5.33%, 73.75%, 18.88%, 11.9%, and 20.18% lower than Model-9, Model-19, Model-3, Model-11, and Model-17, sequentially. In Group-II, the NS (0.029) and a20-index (0.229) values of Model-18 were the highest. Model-17 and Model-19 had negative NS values, which means the performance of these models was very bad. The RMSE and MAPE values of Model-18 were 10.705 MPa and 77.912%, respectively. Based on all the performance metrics, it can be concluded that the performance of Model-18 was good in Group-II models.

**Figure 9:**
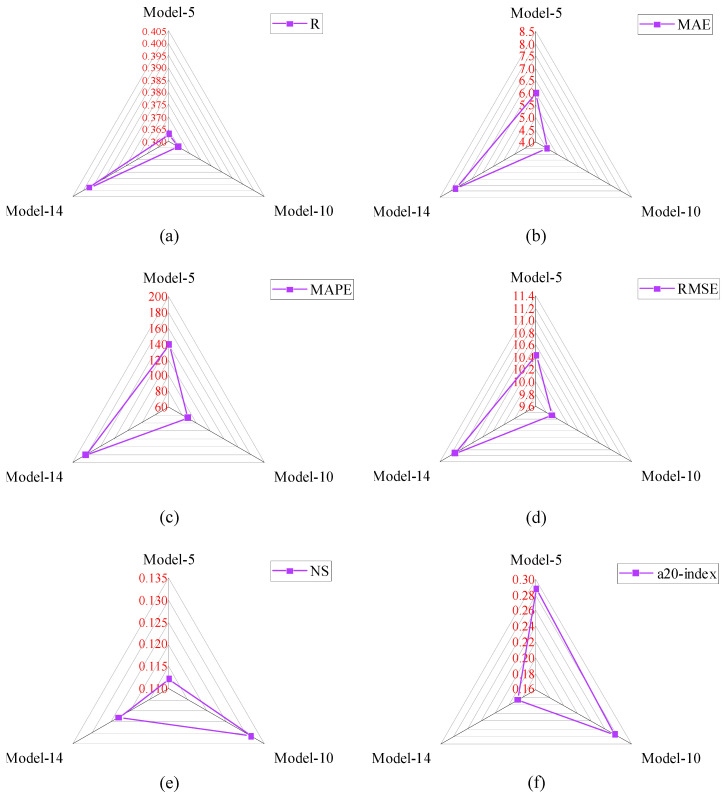
Comparison of performance indices of Group-III models; (**a**) R, (**b**) MAE, (**c**) MAPE, (**d**) RMSE, (**e**) NS, (**f**) a-20 index.

The comparison of performance indices of Group-III models is displayed in Figure 9. In Group-III models, the R-values were between 0.30 and 0.40. Therefore, only three analytical models have fallen into the group. The R-value of Model-14 was 9.37%, and 9.07% higher than Model-5 and Model-10, respectively. The MAE, RMSE, and MAPE values of Model-10 were 4.514 MPa, 9.888 MPa, and 87.517%, respectively. The MAE value of Model-10 was 24.57% and 42.08% lower than Model-5 and Model-14, respectively. Similarly, the RMSE value of Model-10 is 5.21% and 11.17% lower than Model-5 and Model-14, respectively. In addition, the MAPE value of Model-10 was 37.24% and 51.87% lower than Model-5 and Model-14, respectively. The NS value of Model-10 was also greater than other Group-III models. Therefore, based on the analyzed results, it can be summarized that the performance of Model-10 was good compared with other models. The values of all the performance metrics are reported in Table 5.

**Figure 10:**
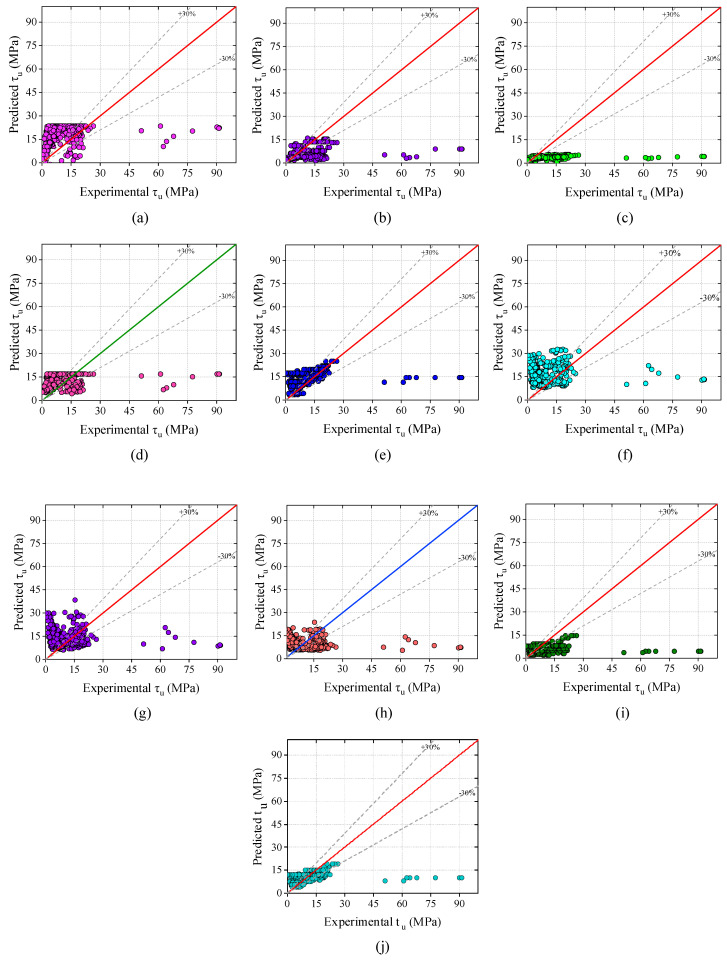

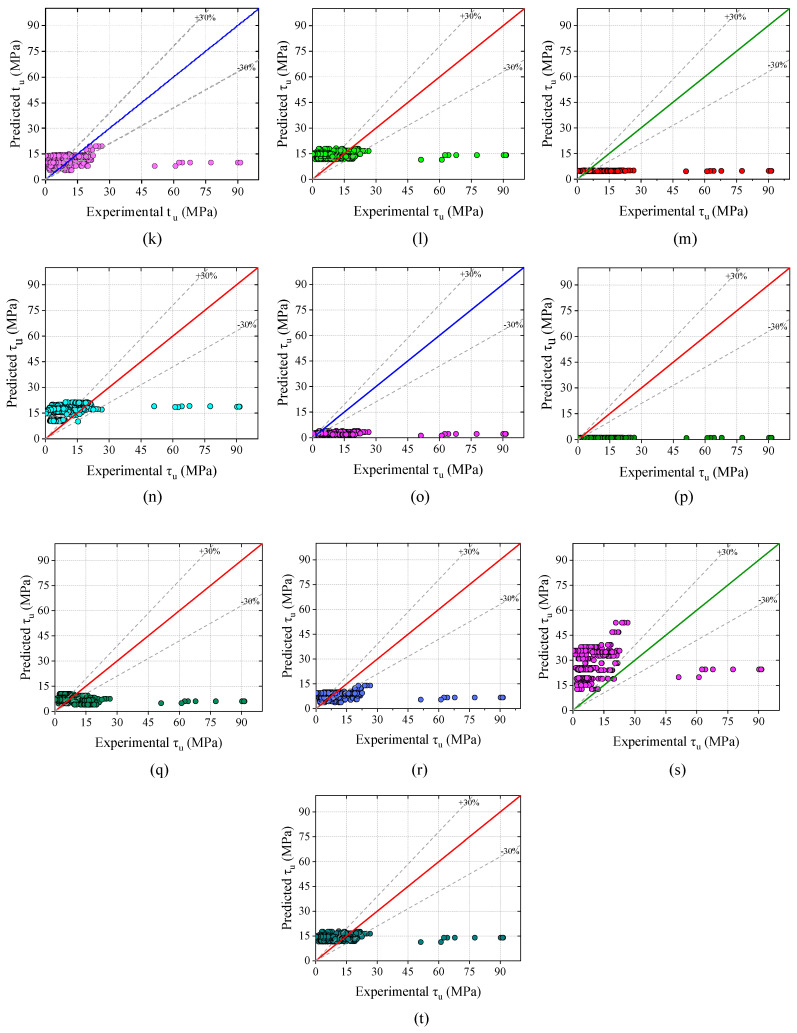
Experimental vs. predicted values of testing samples; (**a**) Model-1, (**b**) Model-2, (**c**) Model-3, (**d**) Model-4, (**e**) Model-5, (**f**) Model-6, (**g**) Model-7, (**h**) Model-8, (**i**) Model-9, (**j**) Model-10, (**k**) Model-11, (**l**) Model-12, (**m**) Model-13, (**n**) Model-14, (**o**) Model-15, (**p**) Model-16, (**q**) Model-17, (**r**) Model-18, (**s**) Model-19, (**t**) Model-20.

Figure 10 indicates the relationship between experimental and predicted values for each of the analytical models. In all the cases, the scatter plots show the poor performance of all the analytical models.

### 5.2. Results of ANN and SVM Models

**Figure 11:**
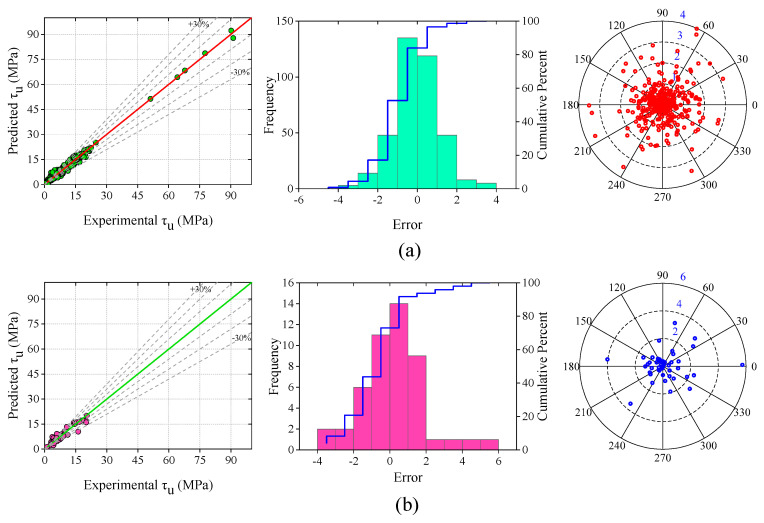

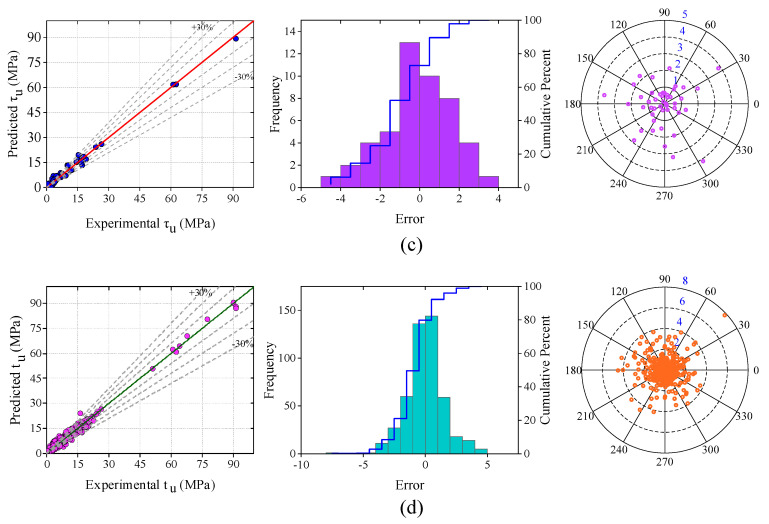
Scatter plot, frequency distribution, and absolute error of proposed ANN model; (**a**) training dataset, (**b**) validation dataset, (**c**) testing dataset, (**d**) overall dataset.

In the development of the ANN model, the dataset was split into three parts, namely training (380 samples), validation (48 samples), and testing (48 samples) datasets. Based on the R and MSE values, thirteen neurons in the hidden layer were selected to build the ANN model. The analyzed results of the ANN model are given in Table 6. The R-values of the training, validation, and testing datasets were 0.994, 0.948, and 0.995, respectively. The overall R-value of the ANN model was 0.99, and the MAE, RMSE, MAPE, NS, and a20-index values of the whole dataset were 1.091 MPa, 1.495 MPa, 17.879%, 0.98, and 0.761, respectively. Figure 11 depicts the scatter plot, frequency distribution, and absolute errors of the developed ANN model. In Figure 11a, the range of the majority of errors (ANN-training) fell between −4 and +4 MPa. The absolute error plot is illustrated on the right side of Figure 11a. The maximum value of the absolute error was 4 MPa. Most of the BS values were within the error range of 2 MPa.

Figure 11b provides the scatter plot, frequency distribution, and absolute errors of the validation dataset. In the scatter plot, almost all the values fell into the error range of −30% to +30%. The frequency distribution of the errors is also shown, which were in the range of –4 MPa to +6 MPa. Meanwhile, Figure 11c displays the scatter plot, frequency distribution, and absolute errors of the testing dataset. The frequency distribution range of the errors, in this case, was between −5 MPa and +4 MPa. The absolute error plot represents that the majority of the error values of BS were within 1 MPa.

The scatter plot, frequency distribution, and absolute errors of the whole dataset are depicted in Figure 11d. The majority of the error values in the frequency distribution plot were in the range of −5 MPa to +5 MPa. In the ANN-all dataset, the absolute error plot indicates that the majority of the error values of BS were within 4 MPa.

**Figure 12:**
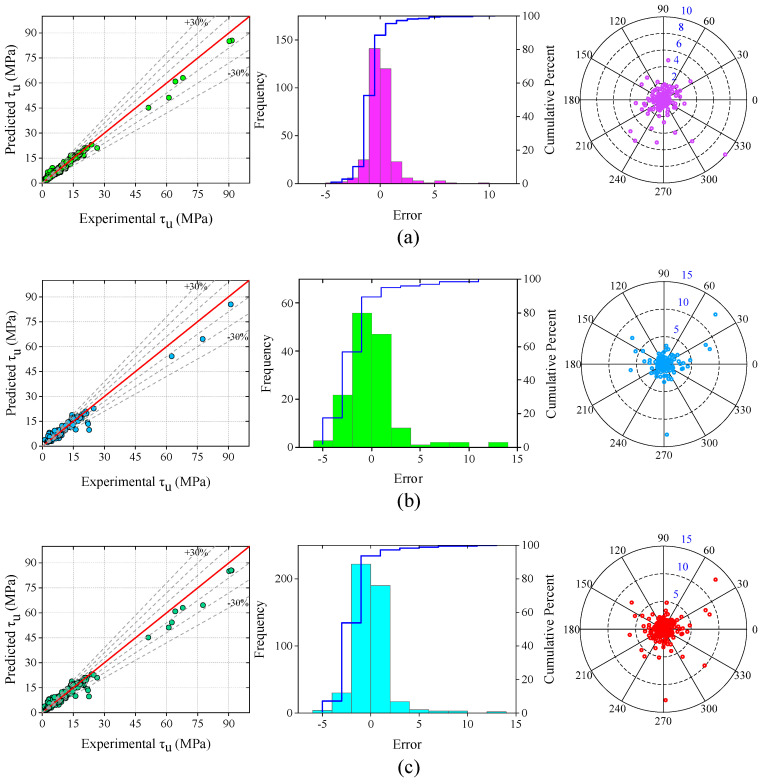
Scatter plot, frequency distribution, and absolute error of proposed SVM model; (**a**) training dataset, (**b**) testing dataset, and (**c**) overall dataset.

The values of all the performance metrics for training, testing, and all datasets of the ANN and SVM models are listed in Table 6. The scatter plot of the developed SVM model with the frequency distribution and absolute error is demonstrated in Figure 12. The R-values of the SVM training, testing, and all datasets were 0.993, 0.979, and 0.989, respectively. Most of the error values in the frequency distribution plot were in the range of −5 MPa to +10 MPa, −5 MPa to 15 MPa, and −5 MPa to +15 MPa for training, testing, and all datasets of the SVM model, respectively. The majority of the BS values in the absolute error plot was within 5 MPa.

### 5.3. Discussion

In the current section, the comparison between the analytical model, proposed ANN and SVM models, and the existing (ML) SVR model is summarized. A Taylor plot is used to show the fitting of the models.

**Figure 13:**
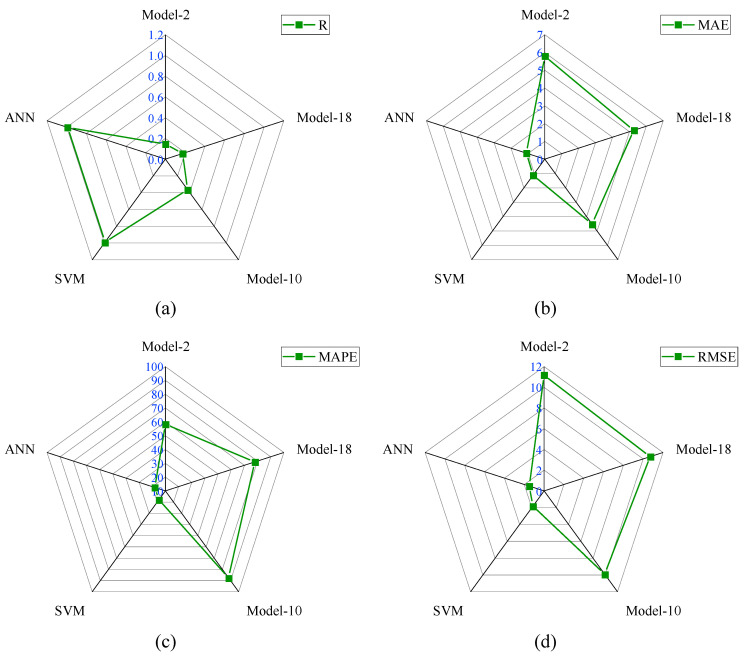

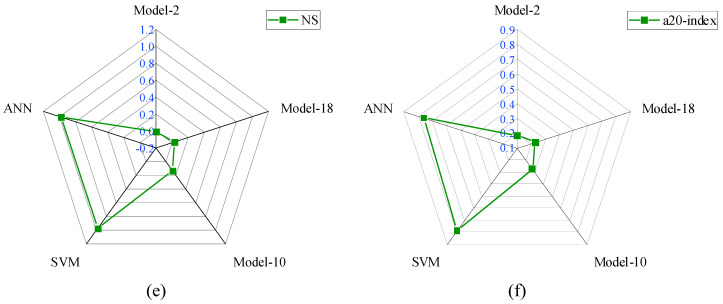
Comparison of performance indices of best-fitted model from each group with proposed SVM and ANN models; (**a**) R, (**b**) MAE, (**c**) MAPE, (**d**) RMSE, (**e**) NS, (**f**) a-20 index.

The best-selected models in Group-I, Group-II, and Group-III were Model-2, Model-18, and Model-10, respectively. The performance indices of the analytical as well as ANN and SVM models are reported in Table 7. In Group-I, II, and III models, the performance of Group-III model was good. The R-value of Model-10 was 149.32% and 109.2% higher than Model-2 and Model-18, respectively. The NS value of Model-10 was 351.72% higher than Model-18. Similarly, the a20-index of Model-10 was 48.65% and 20.09% higher than Model-2 and Model-18, respectively. The MAE and RMSE values of Model-10 were 4.514 MPa and 9.888 MPa, which were the lowest among all the analytical models. The R-value of the ANN model was 0.99, which was 171.98% and 0.1% higher than the R-value of Model-10 and SVM model, respectively. The values of other performance metrics such as MAE, RMSE, MAPE, NS, and a20-index of the ANN model were 1.091 MPa, 1.495 MPa, 17.879%, 0.980, and 0.761, respectively. Similarly, the MAE, RMSE, MAPE, NS, and a20-index of the SVM model were 1.120 MPa, 1.814 MPa, 18.438%, 0.971, and 0.786, respectively. The values of MAE, MAPE, and RMSE of the developed ANN and SVM models were smaller than the error performance indices of Model-10. In addition, the NS and a20-index of the ANN model had higher values compared with Model-10 values. The comparison of the performance indices of the best-fitted model from each group with the proposed SVM and ANN models is presented in Figure 13.

Mousavi et al. [7] used MLP, RBFNN, and SVR algorithms to estimate the steel-to-concrete BS. The SVR model outperformed the other ML models. The R-values of training, testing, and all the datasets were 0.9818, 0.9618, and 0.9772, sequentially. The RMSE values of training, testing, and all the datasets were 3.101 MPa, 3.726 MPa, and 5.372 MPa, respectively.

The R-value of the proposed ANN model was 1.31% higher than the model of Mousavi et al. [7]. The RMSE of the ANN model was 72.17% lower than Mousavi et al. [7] model; this means that the developed ANN model has more accuracy than the existing ML models.

**Figure 14:**
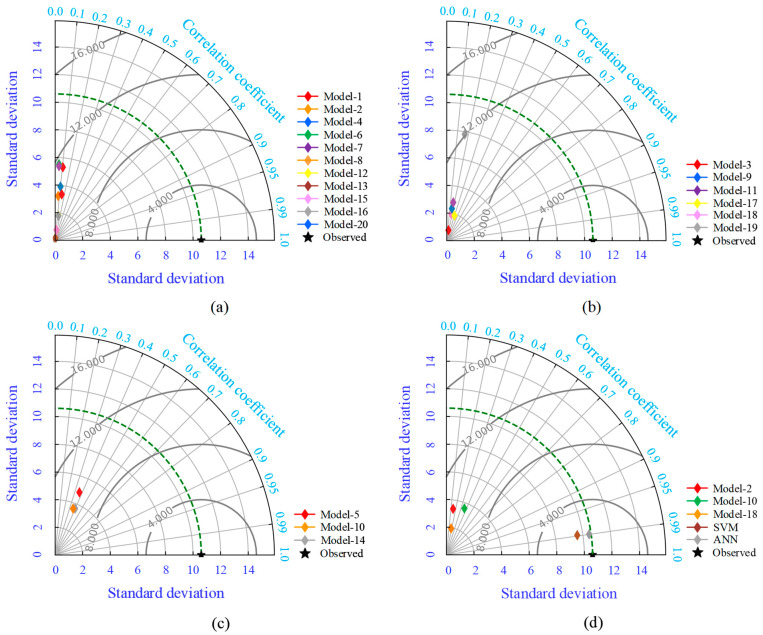
Taylor diagram for all groups; (**a**) Group-I, (**b**) Group-II, (**c**) Group-III, (**d**) Best model from each group, and proposed SVM and ANN models.

In essence, the Taylor diagram is a form of mathematical graphic representation where the baseline indicates that the R-value is one, and the RMSE value is zero, as displayed in Figure 14.

The green dotted line represents the values of the original experimental dataset. If the proposed model patterns are close to the observed patterns, it means that they are similar in terms of their standard deviation, their correlation value is high, and their RMSE value is close to zero. In Figure 14a, all the models in Group-I fell into the standard deviation range of 0.061 to 5.515 and the R-range of 0.053 to 0.146. The overall performance of Group-I models was very poor. Moreover, as illustrated in Figure 14b,c, the performance of Group-II and III models was also poor. The ANN model is directly over the green dotted line in Figure 14d, while the rest of the analytical models from other groups are located far from the green dotted line, except the SVM model. The SVM model is inside the green dotted line because its standard deviation value was lower. The graphical representation of the Taylor diagram also confirms the reliability and accuracy of the developed ANN model.

### 5.4. Relative Importance

**Figure 15:**
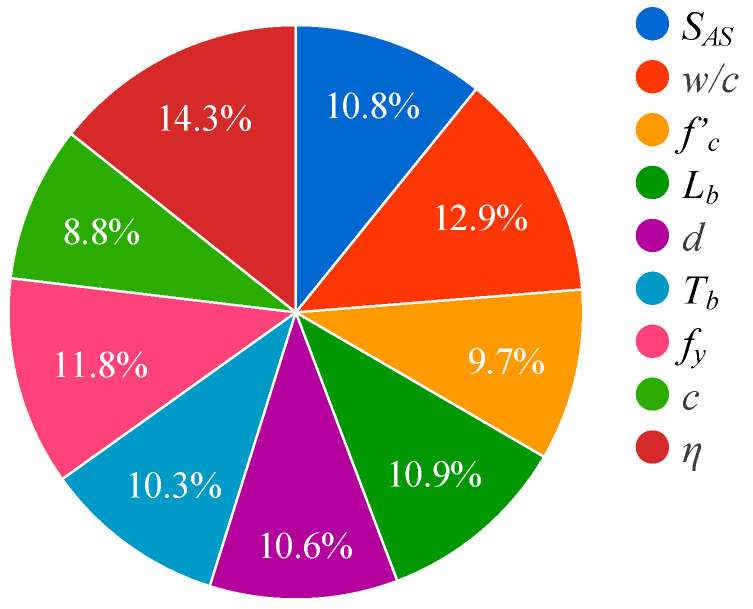
Influencing parameters.

The influence of each input parameter on BS of steel-to-concrete is indicated by the relative importance diagram in Figure 15. According to Figure 15, *η* had the maximum influence on the output (14.3%) which was followed by *w*/*c* (12.9%), *f_y_* (11.8%), *L_b_* (10.9%), *S_AS_* (10.8%), *d* (10.6%), *T_b_* (10.3%), and *f’_c_* (9.7%). The least influenced parameter was c with 8.8% effect on BS.

### 5.5. ANN Formulation

The mathematical expression to calculate the steel-to-concrete BS is expressed in Equation (16). These numeric values are the weights of the output layer, while −1.0047 is the bias of the output layer. The values of the coefficients R_1_ to R_13_ can be obtained from Equation (17).

(16) $$\begin{array}{cc} \tau_{u}=-0.4089R_{1} & +\;0.6782R_{2}-0.3081R_{3}+0.2883R_{4}+0.5820R_{5}-0.2998R_{6}\\ & +\;0.2018R_{7}-1.5947R_{8}-0.2597R_{9}-0.2323R_{10}+0.1298R_{11}\\ & -\;1.6161R_{12}+0.3285R_{13}-1.0047 \end{array}$$

In Equation (17), the first matrix shows the weights of the hidden layer, and the biases are added after the multiplication of normalized input parameters.

(17) $$\left[\begin{array}{c} R_{1}\\ R_{2}\\ R_{3}\\ R_{4}\\ R_{5}\\ R_{6}\\ R_{7}\\ R_{8}\\ R_{9}\\ R_{10}\\ R_{11}\\ R_{12}\\ R_{13} \end{array}\right]=tansig\left[\left(\begin{array}{ccccccccc} 0.3548 & 1.3683 & 1.9614 & -0.4693 & 0.5732 & -4.0172 & 1.9138 & -0.9163 & 1.6698\\ -2.1282 & 0.5958 & 1.4431 & 0.1272 & 0.6748 & -2.1756 & 0.4447 & 0.8862 & 1.6886\\ -1.9044 & 0.5792 & 0.3831 & -3.5589 & 2.0871 & -1.4241 & 1.5820 & 0.1178 & -1.5146\\ 2.5938 & 5.4569 & 1.6255 & -1.6859 & -0.2433 & -1.8327 & -0.0756 & -0.1174 & -2.8664\\ -0.7718 & 0.5722 & -0.3504 & -1.6857 & 0.2004 & 0.9301 & -1.4075 & -0.6732 & -1.2756\\ 0.8357 & 0.7169 & -1.3911 & 4.9729 & 2.0713 & -1.2740 & 0.5758 & -1.7130 & -2.9904\\ 3.8616 & -3.5831 & -1.3878 & -2.0511 & -2.7404 & 0.0287 & -0.5472 & -2.7348 & 2.8703\\ 1.7362 & 0.1706 & -0.6307 & -2.0202 & -2.7355 & 1.3942 & -4.5330 & -0.4639 & 0.8832\\ -1.5375 & 2.0659 & 1.3849 & 0.2211 & -1.8042 & -0.9373 & 1.3450 & 0.7040 & -2.5603\\ -0.5074 & -2.8292 & 0.5880 & -0.6422 & -2.7381 & 1.6172 & -0.4515 & 1.4407 & 1.5384\\ 0.1929 & -1.8666 & 3.2991 & 1.5678 & 1.2715 & -1.0335 & -2.6712 & -4.0505 & 1.0943\\ -0.9449 & -2.1596 & 1.0107 & 0.3392 & -0.2604 & -0.0796 & 1.2981 & 1.1527 & 0.9073\\ -1.0272 & 0.5789 & 1.0876 & -0.2742 & -1.2915 & -0.2745 & 1.8304 & 0.5187 & -2.0369 \end{array}\right)\right]\times \left(\begin{array}{c} S_{AS}\\ w/c\\ f_{c}^{\prime }\\ c\\ L_{b}\\ d\\ T_{b}\\ f_{y}\\ \eta \end{array}\right)+\left(\begin{array}{c} 0.08318\\ -1.3239\\ -0.0840\\ 2.0416\\ 2.7756\\ -1.2344\\ -0.1371\\ 0.2945\\ -0.1330\\ 1.9774\\ -1.2595\\ -2.1739\\ -0.3519 \end{array}\right)$$

## 6. Conclusions

This study developed an ML model to estimate BS between steel surface and concrete. The ANN and SVM models were developed using 476 experimental datasets with input parameters such as *S_AS_*, *w/c*, *f ’_c_*, *c*, *L_b_*, *d*, *T_b_*, *f_y_*, and *η*. Based on the analyzed results, the following conclusions are drawn:

- Based on the R-value, twenty analytical models were divided into three groups Group-I, Group-II, and Group-III with R-ranges of 0 to 0.15, 0.15 to 0.30, and 0.30 to 0.40, respectively. Model-2 performed well in Group-I, with an R-value of 0.146. Similar to this, Model-18 performed well in Group II with an R-value of 0.174, while Model-10 performed well in Group III with an R-value of 0.364.
- The R-value of Model-10 was 149.32% and 109.2% higher than Model-2 and Model-18, respectively. The NS value of Model-10 was 351.72% higher than Model-18. Similarly, the a20-index of Model-10 was 48.65% and 20.09% higher than Model-2 and Model-18, respectively.
- The MAE and RMSE values of Model-10 were 4.514 MPa and 9.888 MPa, which were lowest among all the analytical models. In the analytical models, the performance of Model-10 was good compared with Group-I, II, and III models.
- The R-value of the ANN model was 0.99, which was 171.98% higher than the R-value of Model-10. The values of other performance metrics such as MAE, RMSE, MAPE, NS, and a20-index were 1.091 MPa, 1.495 MPa, 17.879%, 0.980, and 0.761, respectively. The values of MAE, MAPE, and RMSE of the ANN model were smaller than the error performance indices of Model-10. In addition, the NS and a20-index of the SVM and ANN models had higher values than Model-10 values.
- Sensitivity analysis revealed that, as compared with other parameters, the corrosion level had the greatest influence on BS.
- Based on all the analyzed results as well as a graphical representation, it can be concluded that the performance of the ANN model was good, and the developed ML-based ANN model could effectively be used to predict BS of concrete to steel.

In future work, nature-inspired, as well as other ML algorithms, can be used to enhance the accuracy of the proposed model. The collection of more datasets, as well as increasing the range of input and output parameters, also improve the reliability and applicability of this model.

## Figures and Tables

**Table 1:** Summary of ML models used to estimate BS.

S. No.	References	Input Parameter	AI Method Used	Bond Range (MPa)	R (Best Method Value)
1	Golafshani et al. [26]	*f ^′^_c_*, *c*, *A_v_/S*, *R_r_*, *ρ*, *l_s_*	ANN, FL	1.521–8.994	0.993 (FL)
2	Güneyisi et al. [13]	*f ^′^_c_*, *c*, *T_b_*, *d*, *L_b_*, *η*	GEP, ANN	1.3–31.7	0.96 (ANN)
3	Yan et al. [27]	*d*, *c/d*, *L_b_/d*, *d*, *Surf*, *Pos*, *Surf/T_r_*, *√f ^′^_c_*	ANN, GA	2.4–24.52	0.945 (ANN-GA)
4	Yartsev et al. [23]	*f ^′^_c_*, *d*, *η*, *A_d_*, *S_d_*, *Surf*	ANN	1.3–31.7	0.94721 (ANN)
5	Concha and Oreta [28]	*f ^′^_c_*, *L_b_*, *d*, *c*, *UPV*, *η*, *f_t_*, *C_s_*	ANN	Average = 6.591	0.957 (ANN)
6	Yartsev et al. [29]	*f ^′^_c_*, *f_t_*, *A_d_*, *Surf*, *T_b_*, *E_c_*	ANN	—	0.947 (ANN)
7	Bolandi et al. [30]	*Surf*, *Pos*, *d*, *c/d*, *L_b_/d*, *f ^′^_c_*	MGGP	0.76–21	0.961 (MGGP)
8	Hoang et al. [31]	*f ^′^_c_*, *c*, *T_b_*, *d*, *L_b_*, *η*	LSSVR, DFP	1.3–31.7	0.9505 (DFP-LSSVR)
9	Alizadeh et al. [20]	*Surf*, *Pos*, *d*, *c/d*, *L_b_/d*, *f ^′^_c_*, *A_tr_/Snd*	ANFIS, ANN, GMDH	1.64–22.34	0.988 (ANFIS)
10	Concha and Oreta [32]	*f ^′^_c_*, *f_t_*, *L_b_*, *d*, *c*, *UPV*	ANN	Std. deviation = 6.376	0.981 (ANN)
11	Bseiso [24]	*η*, *c*, *f ^′^_c_*	ANN-ReLU, ANN-S	1.3–31.7	0.983 (ANN-ReLU)
12	Ben Seghier et al. [22]	*f ^′^_c_*, *c*, *T_b_*, *d*, *L_b_*, *η*	MLP, RBFNN, GEP	1.3–31.7	0.97 (MLP-LMA)
13	Concha and Oreta [17]	*f ^′^_c_*, *L_b_*, *c/d*, *UPV*	ANN	6.029–30.922	0.927 (ANN)
14	Shahri and Mousavi [33]	*f ^′^_c_*, *d*, *Surf*, *L_b_/d*, *c/d*, *E_s_/E_FRP_*, *A_tr_/Snd*	M5 model tree, MARS, KSM	1.06–6.73	0.969 (MARS)
15	Rahman and Al-Ameri [18]	*f ^′^_c_*, *c/d*, *L_b_/d*, *L_b_*, *d*, *C_t_*	ANN	2.32–14.15	0.963 (ANN)
16	Farouk and Jinsong [34]	*f ^′^_c_*, *UHPC*, *C_m_*, *S_t_*, *I_m_*	SVM, MLR, ANN	0.5–8.85; 1.19–41.2	0.979 (SVM) (Splitting); 0.925 (SVM) (Slant shear)
17	Mousavi et al. [7]	*f ^′^_c_*, *c*, *d*, *L_b_*, *f_y_*, *N_s_*, *A_s_*, *η*	MLP, ANN, RBFNN, SVR	0.19–91.42	0.977 (SVR)
18	Amin et al. [21]	*d*, *f ^′^_c_*, *c/d*, *L_b_/d*	GEP	0.76–21	0.963 (GEP)
19	Farouk et al. [19]	*d*, *f ^′^_c_*, *f_y_*, *d*, *L_b_*, *c*	MLR, SVM, PANN, IEPANN	11.1–71.79	0.973 (IEPANN)

**Table 2:** Details of collected database.

S. No.	References	No. of Specimens	Input Parameters									Output
			*S_AS_* (mm^2^)	*w/c*	*f ′_c_* (MPa)	*L_b_* (mm)	*d* (mm)	*T_b_*	*f_y_* (MPa)	*c* (mm)	*η* (%)	*τ_u_* (MPa)
1	Wei-Liang and Yu-xi [35]	27	10,000	0.55	22.13	80	12	1–2	389–428	44	0.12–9.99	1.4–11.34
2	Horrigmoe et al. [36]	32	80,424.77	0.68	30	160	25	2	500	147.5	0–6.82	3.89–11.91
3	Chung et al. [10]	40	30,000	0.58	28.3	39	13	2	526.8	68.5	0–8.8	9.4–20.1
4	Yalciner et al. [9]	57	22,500	0.4–0.75	23–51	50	14	2	458	15–45	0–18.75	1.8–21.7
5	Zhao et al. [37]	11	22,500	0.36	38.4–41.9	100	18	2	357.5	66	0–6.18	1.18–6.39
6	Choi et al. [12]	9	22,500	0.4–0.6	21–32	100	25	2	524	62.5	0–9.98	51.23–91.42
7	Coccia et al. [8]	10	22,500	0.7	29	70	12	2	507.5	69	0–3.92	4.7–14.13
8	Ma et al. [11]	33	22,500	0.48	24.81	100	18–22	1–2	258.68–373.64	30	0–10.41	1.83–9.05
9	Yalciner and Marar [38]	156	40,000	0.63–0.79	24–38	220–270	14	4	459	30–45	0–4.3	2.134–9.167
10	Hou et al. [39]	42	22,500	0.45	45.5	48–112	16	2	456	48.07–50.27	0–15.25	10.05–21.28
11	Mak et al. [40]	9	8992.02	0.6	22.8–30.7	50	10	2	530	48.5	0–22.9	10.1–17.9
12	Tariq and Bhargava [41]	37	5026.55–31,415.93	0.4	35	40–100	8–20	2	550	36–90	0–16	0.19–22.59
13	Vuong et al. [42]	12	40,000	0.38–0.48	24.6–44.1	60	12	2	400	94	0–12.93	13.81–26.51
14	Lu et al. [43]	1	14,400	0.42	52.6	80	15.2	3	1828	52.4	0	11.2

**Table 3:** Statistical parameters of collected database.

Parameter	Symbol Used	Unit	Minimum	Maximum	Mean	Std.
Concrete	*S_AS_*	mm^2^	5026.55	80,424.77	31,715.84	16,660.00
	*w/c*	^_^	0.36	0.79	0.59	0.14
	*f ′_c_*	MPa	21.00	52.60	31.78	8.36
	*c*	mm	15.00	147.50	52.39	30.82
	*L_b_*	mm	39.00	270.00	132.17	84.75
Reinforcement Bar	*d*	mm	8.00	25.00	15.23	3.83
	*T_b_*	^_^	1.00	4.00	2.59	1.01
	*f_y_*	MPa	258.68	1828.00	463.93	86.39
	*η*	%	0.00	22.90	3.41	4.05
BS	*τ_u_*	MPa	0.19	91.42	10.01	10.60

**Table 4:** Details of analytical models.

Model	References	Formulation	Remarks
Model-1	Cabrera [49]	$f_{bo}=23.478-1.313\;C$	$f_{bo}$ = BS (MPa), *C*_=_ Corrosion level (%)
Model-2	Lee et al. [50]	$\tau_{max}=\{\begin{array}{c} 0.34f_{c}^{\prime }-1.93,\;\Delta w<2.5\%\\ 5.21\;e^{-0.0561\eta },\;\Delta w\geq 2.5\%\; \end{array}$	$\tau_{max}$ = BS (MPa), η = Corrosion percentage (%), $f_{c}^{\prime }$ = CS (MPa)
Model-3	Stanish et al. [51]	$\frac{\tau_{bu}}{\sqrt{f_{c}^{\prime }}}=0.77-0.027\;X_{p}$	$\tau_{bu}\;$ = BS (MPa), $X_{p}$ = Corrosion percentage (%), $f_{c}^{\prime }$ = CS (MPa)
Model-4	Chung et al. [10]	$\{\begin{array}{c} U_{b}=16.87\;for\;C_{o}\leq 2.0\\ U_{b}=24.7\;C_{o}{}^{-0.55}\;for\;C_{o}>2.0 \end{array}$	$U_{b}$ = BS (MPa), $C_{o}$ = Corrosion percentage (%)
Model-5	Aslani and Nejadi [52]	For plain bars, $\tau_{max}=\left[0.7{\left(\frac{c}{d_{b}}\right)}^{0.6}+4\;\left(\frac{d_{b}}{l_{d}}\right)\right]{\left(f_{c}^{\prime }\right)}^{0.23}$ For deformed bars, $\tau_{max}=\left[0.679\;{\left(\frac{c}{d_{b}}\right)}^{0.6}+3.88\;\left(\frac{d_{b}}{l_{d}}\right)\right]{\left(f_{c}^{\prime }\right)}^{0.55}$	$\tau_{max}$ = BS (MPa), *c* = Concrete cover (mm), $d_{b}$ = Diameter of bars (mm), $l_{d}$ = Bond length (mm), $f_{c}^{\prime }$ = CS (MPa)
Model-6	Yalciner et al. [9]	For uncorroded specimens, $\tau_{bu}=-2.7143+0.3621\;f_{c}^{\prime }+2.3296\left(\frac{c}{D}\right)$ For corroded specimens, $\tau_{bu}=0.40551\;f_{c}^{\prime }-0.25306\;\left(\frac{c}{D}\right)+0.97926\;C_{L}$ (R^2^ = 0.98)	$\tau_{bu}$ = BS (MPa), *c* = Concrete cover (mm), *D* = Diameter of bars (mm), $f_{c}^{\prime }$ = CS (MPa), $C_{L}$ = Corrosion level (%)
Model-7	Yalciner and Marar [38]	For uncorroded specimens, $\tau_{HK}=2.9549+0.104\;f_{c}^{\prime }+0.6888\left(\frac{c}{D}\right)$ For corroded specimens, $\tau_{HK}=0.0817\;f_{c}^{\prime }+0.7764\;\left(\frac{c}{D}\right)+1.2647\;C_{L}+3.3657$	*c* = Concrete cover (mm), *D* = Diameter of bars (mm), $f_{c}^{\prime }$ = CS (MPa), $\tau_{HK}$ = BS (MPa), $C_{L}$ = Corrosion level (%)
Model-8	Yalciner and Marar [38]	For uncorroded specimens, $\tau_{HKS}=3.3679+0.1079\;f_{c}^{\prime }+0.0737\left(\frac{c}{D}\right)$ For corroded specimens, $\tau_{HKS}=0.0809\;f_{c}^{\prime }+0.0251\;\left(\frac{c}{D}\right)+0.7568\;C_{L}+4.0116$	$\tau_{HKS}$ = BS (MPa), c = Concrete cover (mm), D = Diameter of bars (mm), $C_{L}$ = Corrosion level (%), $f_{c}^{\prime }$ = CS (MPa)
Model-9	Australian Standard 3600 [53]	$\tau_{u}=0.265\sqrt{{f^{\prime }}_{c}}\left(\frac{c}{d_{b}}+0.5\right)$	—
Model-10	Orangun et al. [54,55]	$\tau_{u}=0.083045\sqrt{{f^{\prime }}_{c}}\;\left[1.2+3.0\left(\frac{c}{d_{b}}\right)+50\left(\frac{d_{b}}{l_{d}}\right)\right]$	—
Model-11	Esfahani and Rangan [56]	$\tau_{u\;}=8.6\left(\frac{\frac{c}{d}+0.5}{\frac{c}{d}+5.5}\right)\;0.55\sqrt{{f^{\prime }}_{c}}$	—
Model-12	CEB-FIP [57]	$\tau_{u}=2.5\sqrt{{f^{\prime }}_{c}}$	$\tau_{u}$ = BS (MPa), $f_{c}^{\prime }$ = CS (MPa)
Model-13	CEB-FIP [57]	$\tau_{u}=7.0\;{\left(\frac{\sqrt{f_{c}^{\prime }}}{25}\right)}^{0.25}$	—
Model-14	Hou et al. [39]	$\tau_{u}=0.335\;\left[-0.124\;\rho_{c}^{2}+1.183\;\rho_{c}+93.504\right]\;{\left(\frac{d}{l_{d}}\right)}^{0.379}$	$\tau_{u}$ = BS (MPa), d = Diameter of bars (mm), $l_{d}$ = Bond length (mm), $\rho_{c}$ = Corrosion ratio (%)
Model-15	Wang et al. [58]	$\tau_{u}=0.09f_{c}^{\prime }-0.655$	$\tau_{u}$$=\mathrm{BS}\;(\mathrm{MPa}),\;f_{c}^{\prime }$ = CS (MPa)
Model-16	Wang et al. [58]	$\tau_{u}=-0.054f_{c}^{\prime }+0.7\sqrt{f_{c}^{\prime }}-1.193$	—
Model-17	Diab et al. [59]	$\tau_{u}=0.08305\sqrt{f_{c}^{\prime }}\;\left[22.8-0.208\left(\frac{c}{d_{b}}\right)-38.212\;\left(\frac{d_{b}}{l_{d}}\right)\right]$	—
Model-18	Eligehausen et al. [60]	$\tau =0.75\sqrt{\frac{c}{d}}{\left(f_{c}^{\prime }\right)}^{0.5}$	—
Model-19	Amini Pishro et al. [61]	$\tau =26.8276\left(\frac{\frac{c}{d}+0.5}{\frac{c}{d}+7.7136}\right)\times 0.55\sqrt{f_{c}^{\prime }}$	—
Model-20	CEB-FIP [57]	$\tau =13.5\sqrt{\frac{f_{c}^{\prime }}{30}}$	$\tau_{u}$ = BS (MPa), $f_{c}^{\prime }$ = CS (MPa)

**Table 5:** Division of models in three groups.

S. No.	Group	Model	R	MAE (MPa)	MAPE (%)	RMSE (MPa)	a20-Index	NS
1	Group-I	Model-6	0.053	9.580	241.660	13.203	0.141	−0.161
2		Model-7	0.054	7.954	209.657	12.480	0.212	−0.289
3		Model-8	0.069	6.503	137.778	11.300	0.191	−0.133
4		Model-4	0.101	7.982	147.153	11.545	0.183	−0.054
5		Model-1	0.107	12.036	222.142	14.462	0.103	−0.084
6		Model-16	0.126	9.008	83.371	13.891	0.006	0.001
7		Model-12	0.138	7.650	174.141	11.218	0.183	0.016
8		Model-15	0.138	7.872	69.634	13.088	0.040	0.009
9		Model-20	0.138	7.550	170.961	11.149	0.181	0.016
10		Model-13	0.139	6.128	59.549	11.777	0.179	0.003
11		Model-2	0.146	5.788	58.224	11.158	0.185	−0.007
12	Group-II	Model-9	0.160	5.550	65.313	11.275	0.223	0.019
13		Model-19	0.168	20.017	422.526	21.771	0.017	−0.075
14		Model-3	0.171	6.477	50.691	12.192	0.191	0.014
15		Model-11	0.171	5.964	125.361	10.549	0.189	0.020
16		Model-18	0.174	5.254	77.912	10.705	0.229	0.029
17		Model-17	0.298	6.582	89.402	11.639	0.151	−0.130
18	Group-III	Model-5	0.363	5.984	139.444	10.432	0.288	0.112
19		Model-10	0.364	4.514	87.517	9.888	0.275	0.131
20		Model-14	0.397	7.794	181.843	11.132	0.187	0.123

**Table 6:** Results of proposed ANN model.

Model	R	MAE (MPa)	MAPE (%)	RMSE (MPa)	a20-Index	NS
ANN-Training	0.994	0.844	13.669	1.127	0.834	0.987
ANN-Validation	0.948	1.213	16.855	1.681	0.667	0.896
ANN-Testing	0.995	1.319	19.235	1.665	0.625	0.990
ANN-All	0.990	1.091	17.879	1.495	0.761	0.980
SVM-Training	0.993	0.896	15.799	1.336	0.826	0.983
SVM-Testing	0.979	1.642	22.937	2.608	0.693	0.948
SVM-All	0.989	1.120	18.438	1.814	0.786	0.971

**Table 7:** Performance indices of the best model from each group I, II, III, and proposed SVM and ANN models.

Group	Model	R	MAE (MPa)	MAPE (%)	RMSE (MPa)	NS	a20-Index
Group-I	Model-2	0.146	5.788	58.224	11.158	−0.007	0.185
Group-II	Model-18	0.174	5.254	77.912	10.705	0.029	0.229
Group-III	Model-10	0.364	4.514	87.517	9.888	0.131	0.275
Existing model	Mousavi et al. [7]	0.977	-	-	5.372	-	-
Proposed model-I	SVM	0.989	1.120	18.438	1.814	0.971	0.786
Proposed model-II	ANN	0.990	1.091	17.879	1.495	0.980	0.761

## Data Availability

Not applicable.

## Nomenclature

SVM	support vector machine	*S_AS_*	surface area of specimen
MLR	multiple linear regression	*w*/*c*	water/cement ratio
ANN	artificial neural network	*f ′_c_*	compressive strength
GEP	gene expression programming	*L_b_*	bond length
RBFNN	radial basis function neural network	*d*	diameter of reinforcement bar
MLP	multilayer perceptron	*T_b_*	type of reinforcement bar
LSSVR	least squares support vector regression	*f_y_*	yield strength of reinforcement bar
DFP	differential flower pollination	*c*	concrete cover
SVR	support vector regression	*η*	corrosion level
MGGP	multi-gene genetic programming	*τ_u_*	bond strength
GA	genetic algorithm	*PF*	pullout force
FL	fuzzy logic	*z_n_*	normalized output of variable
ML	machine learning	*z*	variable of input to be normalized
ReLU	rectified linear unit	*z_min_*	minimum value of input variable z
ANFIS	adaptive neuro-fuzzy inference system	*z_max_*	minimum value of input variable z
GMDH	group method of data handling	*r*	actual output
MARS	multivariate adaptive regression spline	*s*	projected output
MNLR	multiple nonlinear regression	*N*	number of points in data set
KSM	Kriging surrogate model	*N_i_*	input parameter (sum of biases, weights, and normalized inputs)
BPANN	back propagation ANN	*X_i_*	normalized input value
RegTree	regression tree	*W_i(H-O)_*	value of weight from hidden to output layer
PSO	particle swarm optimization	*W_i(I-H)_*	value of weight from input to hidden layer
LMA	Levenberg–Marquardt algorithm	*B_(H-O)_*	value of bias from hidden to output layer
RMSE	root mean square error	*B_(I-H)_*	value of bias from input to hidden layer
MAE	mean absolute error	*f_(I-H)_*	AF that is used from input to hidden layer
MAPE	mean absolute percentage error	*f_(H-O)_*	AF that is used from hidden to output layer
R	correlation coefficient	*R_r_*	relative rib area
NS	Nash-Sutcliffe efficiency index	*A_v_/S*	amount of transverse steel area to spacing ratio
RC	reinforced concrete	*l_s_*	splice length
Std.	standard deviation	*ρ*	splice bar size
MSE	mean square error	*c/d*	ratio of concrete cover to reinforcement bar diameter
BS	bond strength	*L_b_/d*	ratio of bond length to reinforcement bar diameter
A_F_	activation function	*Surf*	reinforcement bar surface treatment
N_s_	number of stirrups	*Pos*	reinforcement bar position/location
A_s_	area of stirrups	*Surf/T_r_*	ratio of reinforcement bar surface to transverse reinforcement bar
C_m_	curing method	*√f ′_c_*	square root of concrete compressive strength
UHPC	ultra-high-performance concrete	*A_d_*	anchorage depth
A_tr_	area of transverse reinforcement bar	*S_d_*	surface dimensions of specimen
E_c_	elastic modulus of concrete	*C_s_*	crack severity of concrete
UPV	ultrasonic pulse velocity	E_s_/EFRP	ratio of elasticity modulus of steel reinforcement bars to that of FRP bars
f_t_	tensile strength of reinforcement bar	IEPSO	improved eliminate particle swarm optimization
S_t_	surface treatment	IEPANN	improved eliminate particle swarm optimization hybridized ANN
i	interface moisture condition	PANN	particle swarm optimization hybridized ANN
C_t_	type of concrete	A_tr_/Snd	ratio of area of transverse reinforcement bar to product (transverse bar spacing, number of developed bars, and bar diameter)
